# Utilization Patterns and Clinical Indications of General Anesthesia in Pediatric Dentistry: A Systematic Review

**DOI:** 10.3390/children13030422

**Published:** 2026-03-19

**Authors:** María Carmona-Santamaría, Davinia Pérez-Sánchez, Juan Ignacio Aura-Tormos, Clara Guinot-Barona, Laura Marqués-Martínez, Esther García Miralles

**Affiliations:** 1 Dentistry Department, Medicine and Health Science Faculty, Catholic University of Valencia, 46001 Valencia, Spain; maria.carmona@ucv.es (M.C.-S.); davinia.perez@ucv.es (D.P.-S.); clara.guinot@ucv.es (C.G.-B.); 2 Stomatology Department, University of Valencia, 46010 Valencia, Spain; juan.aura@uv.es (J.I.A.-T.); m.esther.garcia@uv.es (E.G.M.)

**Keywords:** pediatric dentistry, general anesthesia, early childhood caries, special health care needs, dental rehabilitation, healthcare utilization, public health

## Abstract

**Background:** General anesthesia (GA) plays a key role in pediatric dentistry by enabling comprehensive dental treatment in children who cannot be adequately managed using conventional behavioral techniques, local anesthesia, or sedation. While previous reviews have primarily focused on safety outcomes and adverse events, less attention has been given to patterns of GA utilization and their broader clinical and public health implications. Objective: The objective was to synthesize and critically analyze contemporary evidence on utilization patterns, clinical indications, and treatment characteristics associated with GA in pediatric dentistry and to interpret variability in GA use as a clinical and health-system indicator. **Methods:** A systematic review with qualitative synthesis was conducted in accordance with PRISMA 2020 guidelines. Electronic searches were performed in EBSCOhost, Scopus, and the Cochrane Library to identify observational studies published between 2015 and 2025 reporting clinical data on pediatric dental treatment under GA. **Results:** Twenty-two observational studies met the inclusion criteria. Severe early childhood caries was the most frequently reported indication for GA, followed by behavioral management difficulties and treatment of children with special health care needs. Reported utilization rates varied widely across healthcare systems. **Conclusions:** GA remains an essential modality for managing complex pediatric dental cases; however, variability in utilization appears to reflect differences in preventive access, disease burden, and health-system organization. Interpreting GA use as a healthcare utilization indicator may support improved preventive strategies and policies aimed at reducing repeated GA exposure in vulnerable pediatric populations.

## 1. Introduction

General anesthesia (GA) plays a pivotal role in pediatric dentistry by enabling comprehensive dental care in children who cannot be adequately managed using conventional behavioral techniques, local anesthesia, or conscious sedation [1]. This includes very young children, patients with severe dental anxiety, those requiring extensive oral rehabilitation, and children with special health care needs (SHCN) [2,3]. Furthermore, the global burden of untreated oral diseases in children remains a critical public health concern. According to the World Health Organization (WHO), untreated dental caries in primary teeth significantly affects the quality of life and development of millions of children worldwide [4]. Consequently, clinical guidelines from the American Academy of Pediatric Dentistry (AAPD) emphasize that when pharmacological or non-pharmacological behavior guidance is insufficient, GA is a necessary tool to provide high-quality, safe, and effective dental care [5]. In these populations, GA allows the completion of complex dental procedures in a single session, reducing procedural burden and treatment complexity [6,7].

International clinical guidelines, including those issued by the American Academy of Pediatric Dentistry [8], recognize GA as an appropriate and ethically justified management option when alternative approaches are insufficient to ensure safe and effective care. When administered by trained multidisciplinary teams in appropriate hospital or accredited clinical settings, GA is considered a safe and effective modality that facilitates comprehensive dental rehabilitation while protecting the child’s physical and psychological well-being.

The rapid increase in dental GA utilization in preschool and medically complex cohorts over the last decade, together with persistently high retreatment rates reported in real-world services, justifies an updated synthesis at this time. Although GA is a safe and necessary modality when behavioural and medical factors preclude conventional care, no recent review has jointly characterised (i) utilization-rate heterogeneity as a health-system metric, (ii) contemporary clinical drivers including severe early childhood caries (S-ECC) and specific SHCN diagnostic clusters, and (iii) the durability of restorative planning to reduce repeat GA. This review addresses this gap by integrating multi-regional clinical evidence (2015–2025) to inform fair interpretation of GA workload benchmarking and to support clinicians and policy-makers in strengthening early access and post-GA preventive pathways.

Despite advances in preventive dentistry and minimally invasive approaches, GA continues to be widely used across pediatric dental services worldwide. Severe early childhood caries (S-ECC) is consistently reported as one of the leading clinical drivers for GA-based dental rehabilitation, particularly in preschool-aged children, due to the extent of disease and the practical limitations of cooperation in this age group [9,10]. Observational evidence further indicates that delayed presentation, high baseline caries risk, and socioeconomic vulnerability contribute to the need for single-session full-mouth rehabilitation under GA [11,12].

Beyond caries-related indications, GA is frequently required in children with SHCN and complex medical comorbidities, where cognitive, behavioral, and functional limitations render conventional dental care unsafe or infeasible [13]. Several studies suggest that SHCN populations treated under GA may receive more invasive dental interventions and fewer preventive procedures, reflecting both clinical complexity and potential inequities in access to preventive and routine dental care [14,15]. These findings reinforce the role of GA as a necessary component of comprehensive management rather than an elective option.

The utilization rate of pediatric dental GA varies markedly across regions and clinical settings. Population-level analyses have described increasing utilization of GA in certain contexts, particularly among younger children [12,16]. In parallel, growing evidence highlights that health-system factors—such as availability of hospital-based services, referral pathways, insurance coverage, and preventive infrastructure—contribute substantially to observed differences between countries and regions [17]. In specific high-risk groups, such as children with cleft lip and/or palate, GA requirements appear disproportionately high, reflecting both medical complexity and cumulative treatment needs [18,19].

From a broader public health perspective, reliance on GA for pediatric dental treatment raises important concerns. While GA is generally considered safe when delivered under appropriate conditions, repeated exposure and the need to minimize retreatment remain clinically and ethically relevant issues [20]. High rates of GA utilization have therefore been interpreted as indirect indicators of delayed diagnosis, unmet preventive needs, and underlying social vulnerability affecting pediatric populations [11,12]. Several authors have suggested that excessive reliance on GA may reflect systemic shortcomings in early prevention, caregiver education, and access to timely dental care rather than clinical necessity alone.

Furthermore, increasing attention has been directed toward the long-term outcomes of dental rehabilitation under GA, including recurrence of disease, need for repeat GA, and the durability of restorative strategies. Previous studies have emphasized the importance of restorative choice, particularly the use of stainless steel crowns in high-risk children, as well as structured postoperative preventive programs and caregiver engagement to reduce the likelihood of retreatment [20,21,22]. However, the available evidence remains heterogeneous, and findings are dispersed across diverse healthcare contexts.

Although several reviews have examined general anesthesia in pediatric dentistry, most have primarily focused on safety profiles, adverse events, or anesthetic management considerations. Recent systematic reviews have particularly emphasized perioperative risks and clinical safety outcomes, providing valuable evidence regarding procedural safety but offering limited analysis of how general anesthesia is utilized within pediatric dental care systems. In contrast, less attention has been paid to the interpretation of GA utilization patterns as indicators of disease burden, healthcare organization, and preventive care effectiveness. Furthermore, existing reviews rarely integrate clinical drivers, treatment planning decisions, and long-term implications such as retreatment and repeated GA exposure within a unified public health framework. Therefore, the present review aims not only to summarize clinical indications and treatment characteristics, but also to analyze variability in GA utilization as a health-system and public health indicator, providing an updated synthesis of contemporary evidence (2015–2025) with a broader interpretative perspective.

## 2. Materials and Methods

This study was conducted as a systematic review and reported in accordance with the Preferred Reporting Items for Systematic Reviews and Meta-Analyses (PRISMA) 2020 guidelines.

The PRISMA 2020 checklist is provided as Supplementary Material.

A focused research question was formulated using the PICO framework:

• Population (P): Pediatric patients receiving dental treatment.
• Intervention (I): Dental care under general anesthesia (GA).
• Comparison (C): Not applicable, as this review focuses on characterizing GA use rather than comparing it with other modalities.
• Outcomes (O): GA utilization rates, main clinical indications, dental procedures performed under GA, retreatment and repeated GA, and public health implications.

This systematic review was registered in the PROSPERO International Prospective Register of Systematic Reviews (Registration number: CRD420261329248).

### 2.1. Eligibility Criteria

Original observational studies reporting quantitative clinical data on GA use in pediatric dentistry were included. Eligible studies were published in English or Spanish between 2015 and 2025 and were based on clinical records or institutional databases. Case reports, small case series, editorials, surveys without clinical data, sedation-only studies, and mixed adult–pediatric studies without separable pediatric data were excluded.

Studies published prior to 2015 were excluded from the eligibility criteria; however, earlier publications cited within included studies were used only to contextualize the discussion and were not part of the included dataset.

### 2.2. Search Strategy

A comprehensive and reproducible search strategy was developed in accordance with PRISMA 2020 recommendations. Electronic searches were conducted in EBSCOhost, Scopus, the Cochrane Library, and PubMed/MEDLINE. The strategy combined controlled vocabulary and free-text terms related to general anesthesia and pediatric dental care. In PubMed/MEDLINE, Medical Subject Headings (MeSH) were incorporated when available, including “Anesthesia, General” and “Pediatric Dentistry”, and were combined with keyword searches to capture recently indexed or non-indexed records. Free-text terms included variations and synonyms such as “general anesthesia”, “general anaesthesia”, “dental general anesthesia”, “pediatric dentistry”, “paediatric dentistry”, “child dental care”, “oral rehabilitation”, “treatment under general anesthesia”, “use”, “utilization”, and “indications”. Boolean operators (AND/OR), truncation where appropriate, and database-specific field tags were applied, and search syntax was adapted to the indexing systems and technical requirements of each database to ensure optimal sensitivity and specificity. Searches were limited to studies published between 1 January 2015 and 15 January 2025, and to articles written in English or Spanish. The final search was conducted on 15 January 2025. The complete database-specific search strategies are provided in the Supplementary Material to ensure transparency and reproducibility.

### 2.3. Study Selection

Records were screened by title and abstract, followed by full-text assessment according to predefined eligibility criteria in accordance with PRISMA recommendations.

### 2.4. Data Extraction

Data extraction included study characteristics, population features, indications for GA, procedures performed, utilization estimates, and recurrence or retreatment when reported. Due to substantial heterogeneity in study design, outcome definitions, and reporting formats, a formal quantitative meta-analysis was not considered methodologically appropriate; therefore, results were synthesized using structured qualitative analysis. Data were independently extracted by two reviewers (M.C.S. and D.P.S.), and discrepancies were resolved through discussion and consensus, with involvement of a third senior reviewer (E.G.M.) when necessary.

### 2.5. Risk of Bias Assessment

Risk of bias was independently assessed by two reviewers (M.C.S. and D.P.S.) using the Joanna Briggs Institute (JBI) Critical Appraisal Tools appropriate to each study design [23]. Depending on methodological characteristics, the JBI checklist for cohort studies or the JBI checklist for analytical cross-sectional studies was applied. These tools evaluate domains including clarity of inclusion criteria, validity and reliability of exposure and outcome measurement, identification and management of confounding factors, completeness of follow-up (where applicable), and appropriateness of statistical analysis.

Disagreements between reviewers were resolved through discussion and, when necessary, consultation with a third senior reviewer (E.G.M.). Overall methodological quality was categorized a priori based on the proportion of applicable checklist criteria fulfilled: studies meeting ≥70% of criteria were classified as low risk of bias, those meeting 50–69% as moderate risk, and those fulfilling <50% as high risk. This structured categorization was used to provide a transparent overall appraisal of methodological quality while acknowledging the inherent limitations of retrospective observational designs.

## 3. Results

### 3.1. Study Selection

**Figure 1:**
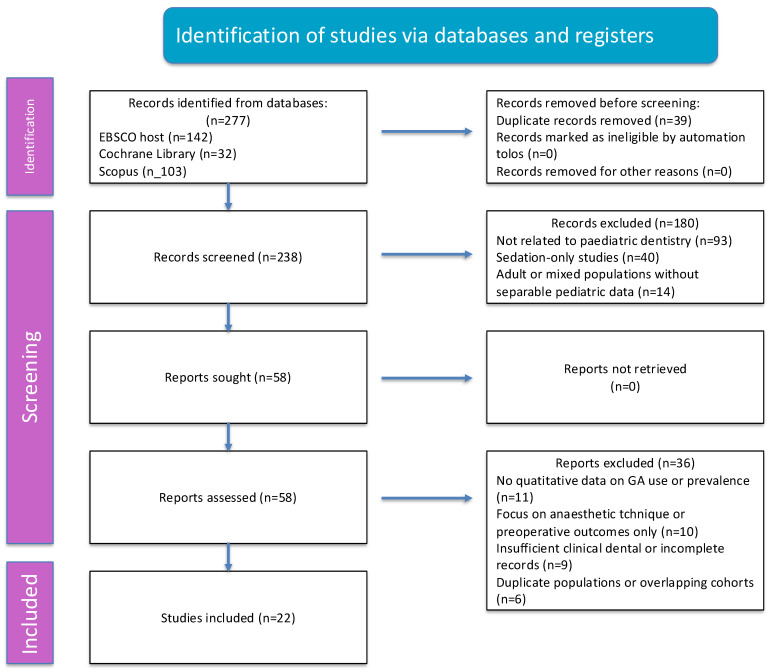
PRISMA 2020 flow diagram illustrating the identification, screening, eligibility, and inclusion of studies in this review.

The database search yielded 277 records after filtering, with 238 unique studies screened following duplicate removal. After full-text assessment, 22 studies met the inclusion criteria and were included in the qualitative synthesis. The study selection process is illustrated in a PRISMA 2020 flow diagram (Figure 1).

### 3.2. Characteristics of Included Studies

Included studies were published between 2017 and 2025 and represented Europe, North America, Asia, the Middle East, and South America. Most were retrospective observational analyses conducted in hospital-based or university-affiliated pediatric dental services [14,19,24,25,26].

The main characteristics of the 22 included studies, including country, study design, population, and primary indications for general anesthesia, are summarized in Table 1.

### 3.3. Main Clinical Indications for General Anesthesia

Across the literature, S-ECC and extensive caries were the most frequently reported indications for GA, particularly in preschool-aged children [9,10,11,12]. Behavioral management difficulties, including young age, dental anxiety, and inability to cooperate, were also consistently cited as major drivers for GA referral [22,27,28]. SHCN populations constituted a substantial subgroup requiring GA due to neurodevelopmental and systemic conditions [13,14,15,16].

### 3.4. Procedures Performed Under General Anesthesia

Treatment under GA commonly involved comprehensive single-session rehabilitation, including restorative procedures, stainless steel crowns (SSCs), pulp therapy, and multiple extractions [22,29,30]. SSCs were repeatedly reported as a durable restorative option, associated with lower retreatment rates compared with direct restorations in high-risk children [21].

### 3.5. Utilization and Geographic Variability

GA utilization rates varied widely across regions and clinical settings. Across the included studies, reported GA utilization rates of dental treatment ranged from below 1% in population-based preventive-oriented systems to over 30% in hospital-based or high-risk pediatric cohorts. Population-based studies described increasing utilization over time in certain health systems [12,17,28]. Environmental and systemic factors, including preventive policies, were implicated in these differences, as shown in studies on treatment variations among different child populations [18].

GA should therefore be interpreted primarily as a healthcare utilization metric rather than a direct epidemiological measure of disease prevalence.

High-risk groups such as children with cleft lip and/or palate demonstrated particularly elevated GA requirements [19].

### 3.6. Recurrence and Retreatment Under General Anesthesia

Repeat GA and retreatment were reported in several studies, with higher rates observed in younger children and high-risk populations [30,31,32,33]. Restorative choices influenced recurrence, with stainless steel crowns (SSCs) consistently associated with greater durability and fewer re-interventions, supporting the reduction in repeated GA exposure [21,22].

Clinical guidelines and reviews focusing on SHCN populations emphasize the importance of preventive strategies to reduce repeated GA exposure [34,35].

Repeat GA was more frequently associated with less durable direct restorative approaches in high-risk primary molars, whereas stainless steel crowns (SSCs) supported improved procedural longevity and fewer re-interventions [21,22,32,34].

### 3.7. Risk of Bias Assessment

Overall methodological quality was predominantly moderate (Table 2), reflecting the retrospective and frequently single-center design of most included studies. One study was assessed as high risk of bias due to limited methodological transparency and small sample size. No studies met criteria for classification as low risk of bias.

## 4. Discussion

### 4.1. Study Aim and Methodological Considerations

The primary aim of this review was to synthesize and critically analyze the current evidence regarding the utilization rate, clinical indications, and treatment patterns associated with the use of general anesthesia in contemporary pediatric dentistry. To achieve this, a methodological approach based on a systematic review was adopted. This methodology enabled a structured synthesis of 22 included studies, integrating qualitative interpretation through systematic evidence synthesis to characterize patterns of GA utilization across different healthcare systems. The results derived from the included studies confirm that GA remains an indispensable modality for managing complex clinical cases, especially in patients with severe early childhood caries (S-ECC). The methodological quality of the included studies was assessed using the Joanna Briggs Institute (JBI) critical appraisal tools appropriate to each study design.

The findings of this review suggest that general anesthesia utilization should be interpreted not only as a clinical management strategy but also as an indicator of healthcare organization and preventive system performance. Within this framework, although GA remains an effective tool for providing comprehensive dental treatment in a single session, its use appears to be influenced by a combination of clinical and non-clinical factors. One of the most significant clinical indications identified across the included studies was severe early childhood caries (S-ECC). Children presenting with extensive decay, frequently involving multiple teeth and associated with pain or infection, often require GA due to treatment complexity and limited cooperation capacity [14,15,16]. The predominance of S-ECC among GA cases therefore reflects an ongoing public health challenge and underscores the need for earlier intervention and strengthened preventive strategies [1,7,10].

### 4.2. Comparison with Previous Reviews

Previous reviews on general anesthesia in pediatric dentistry have predominantly focused on anesthetic safety, perioperative complications, and adverse events, providing essential evidence regarding procedural risk and anesthetic management. However, these studies have offered limited evaluation of how general anesthesia is utilized across pediatric dental care systems or how utilization patterns may reflect broader healthcare and public health dynamics.

Building on this gap, the present review examines variability in GA utilization rates, underlying clinical drivers, treatment planning strategies, and recurrence patterns within a unified interpretative framework. By integrating utilization data with clinical indications and health-system determinants, GA use is interpreted not only as a clinical intervention but also as a healthcare utilization indicator reflecting disease burden, access to preventive care, and structural characteristics of pediatric dental services.

This perspective complements, rather than contradicts, previous reviews by shifting the analytical focus from procedural safety toward system-level interpretation and long-term clinical implications. Such an approach may assist clinicians and policymakers in understanding differences in GA demand across populations and in developing strategies aimed at strengthening early prevention and reducing repeated exposure to general anesthesia.

### 4.3. Interpretation of Main Findings

#### 4.3.1. Clinical Indications for General Anesthesia

The synthesized evidence robustly confirms that severe early childhood caries (S-ECC) remains the predominant clinical driver for GA in pediatric dentistry globally, particularly among preschool-aged children [9,10]. This finding is consistent with broader public health literature that identifies S-ECC as a highly prevalent and impactful disease, often requiring comprehensive rehabilitation due to its rapid progression and association with significant morbidity [20]. The results further underscore that behavioral management difficulties—rooted in young age, severe dental anxiety, or an outright inability to cooperate—constitute a second major pillar of GA indications [22,26]. This aligns with established pediatric dental principles, where a child’s emotional and cognitive readiness is as critical as the technical treatment needs [8,36]. Completing comprehensive dental treatment in a single GA session allows management of extensive disease while reducing the need for multiple treatment visits and repeated exposure to invasive procedures [6,11].

A particularly vulnerable and clinically complex subgroup identified across the included studies comprises children with special health care needs (SHCN) [13,14]. Our synthesis indicates that these patients frequently present with more advanced disease and undergo more invasive procedures under GA. This pattern likely reflects a confluence of factors: inherent medical and behavioral complexities, systemic barriers to accessing routine and preventive dental care, and often delayed diagnosis. These observations resonate with prior literature emphasizing the critical need for tailored dental care pathways and the essential role of GA in providing equitable, comprehensive treatment for this population [2,15].

#### 4.3.2. GA Utilization Rates and Health-System Determinants

The extreme variability in GA utilization rates—ranging from below 1% in population-based preventive systems to over 30% in high-risk or hospital-based cohorts—constitutes a central finding of this review. This wide disparity cannot be attributed solely to differences in clinical need or caries burden. Instead, it appears to be profoundly influenced by health-system and structural determinants, including the organization of referral pathways, insurance coverage models, availability of hospital dental services, and the strength of public health preventive infrastructure [12,16,17]. For instance, the global perspective on early childhood caries management highlights how public health interventions are critical [20].

Such observations position GA utilization not merely as a clinical metric, but as a potential sentinel indicator of broader oral health inequalities, unmet preventive needs, and systemic gaps in pediatric healthcare access.

Consequently, high GA utilization rates, particularly among vulnerable groups such as children with S-ECC or SHCN, should be interpreted primarily as a reflection of barriers to early conventional dental care and upstream prevention, rather than as an epidemiological measure of disease prevalence. This distinction is crucial: GA is a treatment modality, not a disease. Therefore, its variability serves as a healthcare utilization metric that can reveal shortcomings in health policy, resource allocation, and social determinants of health affecting pediatric populations. Monitoring GA rates could thus inform targeted public health interventions aimed at strengthening preventive programmes, improving early access to dental care, and addressing the underlying social vulnerabilities that drive dental disease in children.

#### 4.3.3. Treatment Patterns, Recurrence, and Long-Term Outcomes

Dental rehabilitation under GA is characterized by comprehensive, single-session treatment. The most common procedures include restorative treatments with stainless steel crowns (SSCs) or composites, pulp therapy, and multiple extractions [21,22]. A critical insight from the analyzed evidence is the significant association between restorative choice and long-term outcome. SSCs were consistently associated with lower failure and retreatment rates compared to direct restorations in high-caries-risk children [21], a finding supported by studies specifically advocating for the preferential use of SSCs as a strategy to minimize the need for retreatment under GA [22]. This supports existing clinical guidelines that advocate for SSCs as the restoration of choice for extensive caries in primary molars due to their durability and superior marginal integrity [34]. SSCs reduce repeat GA exposure due to improved procedural longevity in high-risk primary molars.

The issue of recurrence—needing retreatment or repeat GA—emerges as a major clinical and ethical concern, particularly for very young children and those with SHCN [32,33]. Risk factors for recurrence include younger age at first GA, high baseline caries risk, and insufficient postoperative preventive follow-up. These findings powerfully underscore that the GA session itself, while resolving immediate dental pathology, is not a definitive solution. Its long-term success is inextricably linked to the implementation of robust, individualized preventive regimens following rehabilitation. This includes structured recall systems, intensive caregiver education, and dietary counseling, as emphasized in several studies included in this review [11,22].

The substantial heterogeneity observed likely reflects differences in healthcare organization, patient risk profiles, and follow-up duration, reinforcing the interpretation of GA utilization as a system-level outcome influenced by preventive access and disease burden.

#### 4.3.4. Neurodevelopmental and Mental Health Concerns

Evidence has raised concerns regarding potential associations between early or repeated GA exposure and neurodevelopmental outcomes; however, findings are heterogeneous and causality is limited by confounding (e.g., prematurity, SHCN, comorbidity burden). The GAS randomized trial reported no significant neurodevelopmental differences at 2 years after a single exposure compared with awake-regional anesthesia, supporting reassurance for single, well-controlled exposures [7]. Clinically, minimizing repeat GA is best achieved through durable restorative planning, structured recalls, and intensive prevention.

### 4.4. Limitations of the Evidence and Future Directions

The findings of this review should be interpreted in light of the limitations inherent to the available evidence base. The predominance of retrospective, single-center observational studies introduces potential risks of selection bias, residual confounding, and limited external validity. Substantial heterogeneity in study design, population characteristics, definitions of clinical indications, and outcome reporting limited direct comparability across studies and precluded formal quantitative meta-analysis. In addition, follow-up duration was inconsistently reported, restricting robust conclusions regarding long-term recurrence and repeated general anesthesia exposure, including sustained oral health stability and behavioral outcomes.

These limitations delineate priorities for future research. Prospective, multicenter studies with standardized definitions of GA indications, harmonized outcome reporting, and clearly defined follow-up intervals are warranted. Further investigation into preventive integration and optimized referral pathways is needed to ensure timely access to GA when clinically indicated while strengthening upstream preventive strategies within pediatric dental care systems [36].

### 4.5. Clinical and Public Health Implications

From a clinical standpoint, this review reinforces the importance of meticulous case selection for GA, grounded in clear guidelines. It strongly advocates for the use of the most durable restorative options, like SSCs, in high-risk scenarios to minimize retreatment. Crucially, it mandates that every GA episode be viewed as the beginning of an intensive preventive phase, requiring committed follow-up and family engagement.

From a public health perspective, the wide variability in GA use serves as a call to action. High or rising rates of GA should prompt an examination of underlying systemic failures in prevention, early intervention, and access to care. Policy efforts should be directed toward strengthening community-based prevention, improving access to early dental visits, and addressing the social determinants that fuel dental disease in children. Ultimately, the goal is not to eliminate GA—an indispensable tool for complex cases—but to ensure its use is reserved for appropriate clinical indications rather than being a default response to widespread, preventable disease.

## 5. Conclusions

General anesthesia remains a necessary and justified approach for pediatric dental care in selected clinical scenarios, particularly in cases of severe early childhood caries, special health care needs, and behavioral non-cooperation. However, beyond its clinical role, variability in GA utilization patterns may also reflect differences in disease burden, preventive effectiveness, and structural characteristics of pediatric dental care systems. The findings of this review suggest that reliance on GA is influenced not only by individual treatment needs but also by system-level factors, including access to preventive services and organization of care pathways. Reducing repeat GA exposure therefore requires strengthening early prevention, improving timely access to conventional dental care, enhancing caregiver education, and prioritizing durable restorative strategies in high-risk children. Collectively, these measures may contribute to more equitable care delivery and improved long-term oral health outcomes in pediatric populations.

## Figures and Tables

**Table 1:** Characteristics of the included studies.

Study (Author/Year)	Country	Study Design	Study Population	*n*	Main Indications for GA	Main Dental Procedures Under GA
Berry et al. (2017) [2]	USA	Retrospective observational	Pediatric patients	40	Behavioral management difficulties	Full-mouth rehabilitation
Da Silva et al. (2022) [6]	Canada	Retrospective observational	Pediatric patients	570	Severe early childhood caries	Restorations, SSCs, pulp therapy
He et al. (2023) [9]	China	Retrospective cohort	Children with S-ECC	852	Extensive caries	Pulpectomies, restorations
Ibrahim et al. (2023) [10]	Malaysia	Retrospective observational	Pediatric patients	381	Caries, non-cooperation	Restorations, extractions
Edmonds et al. (2019) [11]	USA	Retrospective cohort	Pediatric patients	40	Repeat GA	Mixed procedures
Meyer et al. (2017) [12]	USA	Population-based study	Pediatric population	632,941	System-level utilization	Mixed procedures
Kaviani et al. (2021) [13]	Iran	Cross-sectional	SHCN vs. healthy children	361	SHCN-related limitations	Extractions, restorations
Mallineni (2018) [14]	Hong Kong	Retrospective observational	Children with SHCN	275	Medical and behavioral limitations	Restorations, SSCs
Surtie et al. (2024) [15]	Multicenter	Retrospective cohort	Children with cleft lip/palate	441	Medical complexity	Restorations, extractions
Koberova Ivancakova et al. (2019) [16]	Czech Republic	Retrospective cohort	Children with disabilities	229	Behavioral and cognitive limitations	Extractions, restorations
Shayegan et al. (2025) [17]	Belgium	Retrospective analysis	Pediatric population	4062	Extensive treatment needs; behavioral and medical limitations	Mixed procedures
Bekes et al. (2020) [18]	Germany	Retrospective observational	Pediatric patients	652	Non-cooperation, extensive caries	Restorations, extractions
Karl et al. (2022) [21]	Germany	Retrospective cohort	Pediatric patients	1155	Repeated GA requirement	Full-mouth rehabilitation
Patel et al. (2021) [22]	USA	Retrospective cohort	Pediatric patients	296	Treatment failure	SSCs, restorations
Hieronymus et al. (2024) [26]	Germany	Retrospective observational	Children with SHCN	669	Intellectual disability, neurological disorders	Restorations, extractions
Condori et al. (2023) [27]	Peru	Retrospective observational	Pediatric patients	54	Extensive dental treatment needs	Restorations, SSCs
Weninger et al. (2022) [28]	Canada	Cross-sectional	Pediatric patients	90	High caries burden	Full-mouth rehabilitation
Shukry et al. (2022) [29]	Malaysia	Retrospective observational	Pediatric patients	125	Behavioral difficulties	Full-mouth rehabilitation
Najib et al. (2021) [30]	Bahrain	Retrospective observational	Pediatric patients	281	Extensive treatment needs	Full-mouth rehabilitation
Guidry et al. (2017) [31]	USA	Retrospective observational	Pediatric patients	581	Caries, non-cooperation	Extractions, restorations
Radacsi et al. (2023) [32]	Hungary	Retrospective observational	Pediatric patients	525	Severe caries	Extractions
Rudie et al. (2018) [33]	USA	Retrospective cohort	Pediatric patients	3973	Repeat GA risk	Mixed procedures

**Table 2:** Methodological quality assessment using JBI critical appraisal tools.

Study	Study Design	JBI Tool Applied	Overall Risk of Bias
Berry et al. (2017) [2]	Retrospective observational	JBI Cohort Checklist	Moderate
Da Silva et al. (2022) [6]	Retrospective observational	JBI Cohort Checklist	Moderate
He et al. (2023) [9]	Retrospective cohort	JBI Cohort Checklist	Moderate
Ibrahim et al. (2023) [10]	Retrospective observational	JBI Cohort Checklist	Moderate
Edmonds et al. (2019) [11]	Retrospective cohort	JBI Cohort Checklist	Moderate
Meyer et al. (2017) [12]	Population-based retrospective	JBI Cohort Checklist	Moderate
Kaviani et al. (2021) [13]	Cross-sectional	JBI Cross-sectional Checklist	Moderate
Mallineni et al. (2018) [14]	Retrospective observational	JBI Cohort Checklist	Moderate
Surtie et al. (2024) [15]	Multicenter retrospective cohort	JBI Cohort Checklist	Moderate
Koberova Ivancakova et al. (2019) [16]	Retrospective cohort	JBI Cohort Checklist	Moderate
Shayegan et al. (2025) [17]	Retrospective analysis	JBI Cohort Checklist	Moderate
Bekes et al. (2020) [18]	Retrospective observational	JBI Cohort Checklist	Moderate
Karl et al. (2022) [21]	Retrospective cohort	JBI Cohort Checklist	Moderate
Patel et al. (2021) [22]	Retrospective cohort	JBI Cohort Checklist	Moderate
Hieronymus et al. (2024) [26]	Retrospective observational	JBI Cohort Checklist	Moderate
Condori et al. (2023) [27]	Retrospective observational	JBI Cohort Checklist	High
Weninger et al. (2022) [28]	Cross-sectional	JBI Cross-sectional Checklist	Moderate
Shukry et al. (2022) [29]	Retrospective observational	JBI Cohort Checklist	Moderate
Najib et al. (2021) [30]	Retrospective observational	JBI Cohort Checklist	Moderate
Guidry et al. (2017) [31]	Retrospective observational	JBI Cohort Checklist	Moderate
Radacsi et al. (2023) [32]	Retrospective observational	JBI Cohort Checklist	Moderate
Rudie et al. (2018) [33]	Retrospective cohort	JBI Cohort Checklist	Moderate

## Data Availability

No new data were created or analyzed in this study. Data sharing is not applicable to this article.

## Supplementary Materials

The following supporting information can be downloaded at: https://www.mdpi.com/article/10.3390/children13030422/s1, The PRISMA 2020 checklist.

## Abbreviations

GA	general anesthesia
SHCN	special health care needs
SSCs	stainless steel crowns
S-ECC	severe early childhood caries
