# Supplementary material for: Utilization Patterns and Clinical Indications of General Anesthesia in Pediatric Dentistry: A Systematic Review
Source: Children (Basel). 2026 Mar 19;13(3):422. doi: 10.3390/children13030422 (PMC13025537; doi:10.3390/children13030422)
Supplement: Supplementary file 1 [file children-13-00422-s001.zip › children-4205675-supplementary.pdf]

PRISMA 2020 Checklist

Manuscript title: Utilization Patterns and Clinical Indications of General Anesthesia in Pediatric Dentistry: A Systematic Review

Section and Topic	Item	Checklist item	Location in manuscript
TITLE	1	Identify the report as a systematic review	Title page
ABSTRACT	2	Structured summary including objectives, methods, results, and conclusions	Abstract
INTRODUCTION	3	Describe rationale for the review	Introduction
4	Provide explicit statement of objectives	End of Introduction	
METHODS	5	Eligibility criteria	Section 2.1
6	Information sources	Section 2.2	
7	Search strategy	Section 2.2	
8	Selection process	Section 2.3	
9	Data collection process	Section 2.4	
10	Data items	Section 2.4	
11	Risk of bias assessment	Section 2.5	
12	Effect measures	Not applicable (qualitative synthesis)	
13	Synthesis methods	Section 2.4 (qualitative synthesis justification)	
14	Reporting bias assessment	Addressed in Limitations	
15	Certainty assessment	Not applicable	
RESULTS	16	Study selection	Section 3.1; Figure 1
17	Study characteristics	Table 1	
18	Risk of bias results	Section 3.2	

Section and Topic	Item	Checklist item	Location in manuscript
19	Results of individual studies	Sections 3.3–3.6	
20	Results of syntheses	Sections 3.3–3.6	
21	Reporting biases	Discussed in Section 4.4	
22	Certainty of evidence	Not applicable	
DISCUSSION	23	General interpretation of results	Section 4
24	Limitations of evidence	Section 4.4	
25	Implications for practice and research	Sections 4.5 and Conclusions	
OTHER INFORMATION	26	Registration and protocol	Explained in Section 4.4
27	Support	Title page / declarations	
28	Competing interests	Declarations	
29	Availability of data	Data availability statement	
